# Computational modeling, docking and molecular dynamics of the transcriptional activator ComA bound to a newly-identified functional DNA binding site

**DOI:** 10.1186/1758-2946-6-S1-P30

**Published:** 2014-03-11

**Authors:** Juan C Mobarec, Diana Wolf, Ilka B Bischofs, Peter Kolb

**Affiliations:** 1 Department of Pharmaceutical Chemistry, Philipps University Marburg, Marburg, Hesse, 35032, Germany; 2 Ruprecht-Karls-University Heidelberg, Heidelberg, Germany

## 

Quorum sensing is the mechanism by which bacterial cells communicate to each other in response to changes in cell density. Secreted signaling molecules reach other bacteria and trigger an internal physiological response, like production of degradative enzymes and antibiotics, competence development, sporulation and pathogenesis. In *Bacillus subtilis*, the transcriptional activator ComA regulates several genes of the quorum sensing response. ComA binds to recognition elements (RE) in bacterial promoters, activating transcription. The DNA binding domain of ComA has four α-helices, which contain a helix-turn-helix (HTH) motif. Several promoters in *B. subtilis* have known inverted repeat motifs (RE1 and RE2), where a ComA dimer can bind. However, a third and fourth recognition-like element (RE3 and RE4) in a direct repeat (DR) arrangement have recently been identified downstream of the known ComA box. Currently there is no structural information on how a DR would bind to ComA.

Here, we present computational results supported by experiments that the DR (RE3 and RE4) form a functional domain for recognition of a ComA dimer. Flexible protein-DNA docking was used to get insight into the putative binding mode of ComA bound to the new direct repeat. The lowest-energy docked conformations of the ComA-DNA complex were tested for dynamic stability with explicit molecular dynamics simulations. Clustering of the sampled conformations was used to select a representative structure of the ComA-DR- DNA complex. In our results, the second α-helix of the HTH contributes most of the DNA recognition, binding to the major grooves of the DR-DNA, and interacting mostly with the DNA bases. We pinpoint specific ComA-DNA interactions that may have a key role for recognition and affinity. Furthermore, the physical interaction between ComA and the new DR was demonstrated *in vitro*, and its functionality was confirmed *in vivo*.

Our results strongly support the hypothesis that ComA dimers can bind to direct repeats, and provide an atomistic model for its recognition. Additionally, we suggest specific interactions for fine-tuning of transcription which will be tested experimentally.

